# Surgical Site Infections, Risk Factors, and Outcomes After Liver Transplant

**DOI:** 10.1001/jamanetworkopen.2025.1333

**Published:** 2025-03-21

**Authors:** Peter W. Schreiber, Linard D. Hoessly, Katia Boggian, Dionysios Neofytos, Christian van Delden, Adrian Egli, Michael Dickenmann, Cédric Hirzel, Oriol Manuel, Michael Koller, Simona Rossi, Vanessa Banz, Philippe Compagnon, Philipp Dutkowski, Andreas E. Kremer, Annalisa Berzigotti, Julien Vionnet, Nicolas Goossens, David Semela, Patrizia Künzler-Heule, Christine Bernsmeier, Stefan P. Kuster, Susanne Stampf, Nicolas J. Mueller

**Affiliations:** 1 Department of Infectious Diseases and Hospital Epidemiology, University Hospital Zurich and University Zurich, Zurich, Switzerland; 2 Clinic for Transplantation Immunology and Nephrology, Basel University Hospital, Basel, Switzerland; 3 Division of Infectious Diseases, Infection Prevention and Travel Medicine, Cantonal Hospital St Gallen, St Gallen, Switzerland; 4 Transplant Infectious Diseases Unit, Service of Infectious Diseases, University Hospitals Geneva, University of Geneva, Geneva, Switzerland; 5 Institute of Medical Microbiology, University of Zurich, Zurich, Switzerland; 6 Clinical Bacteriology & Mycology, University Hospital Basel, University of Basel, Switzerland; 7 Department of Infectious Diseases, Inselspital, Bern University Hospital, University of Bern, Bern, Switzerland; 8 Infectious Diseases Service, University Hospital (CHUV) and University of Lausanne, Lausanne, Switzerland; 9 Transplantation Center, University Hospital (CHUV) and University of Lausanne, Lausanne, Switzerland; 10 Department of Visceral Surgery and Medicine, Inselspital, Bern University Hospital, University of Bern, Bern, Switzerland; 11 Division of Abdominal Surgery, Department of Surgery, University Hospitals Geneva, Geneva, Switzerland; 12 Division of Transplant Surgery, Department of Surgery, University Hospitals Geneva, Geneva, Switzerland; 13 Department of Surgery and Transplantation, Swiss HPB Centre, University Hospital Zurich, Zurich, Switzerland; 14 Department of Gastroenterology and Hepatology, University Hospital Zürich, University of Zurich, Zurich, Switzerland; 15 Department of Visceral Surgery and Medicine, Inselspital, University Hospital Bern and University of Bern, Bern, Switzerland; 16 Transplantation Centre and Service of Gastroenterology and Hepatology, Lausanne University Hospital and University of Lausanne, Lausanne, Switzerland; 17 Division of Transplantation, University Hospitals Geneva, Geneva, Switzerland; 18 Division of Gastroenterology & Hepatology, University Hospitals Geneva, Geneva, Switzerland; 19 Division of Gastroenterology and Hepatology, Cantonal Hospital St Gallen, St Gallen, Switzerland; 20 University Centre for Gastrointestinal and Liver Diseases, Basel, Switzerland

## Abstract

**Importance:**

Surgical site infections (SSIs) are one of the most common health care–associated infections. Surgical site infections can have harmful effects in liver transplant (LT) recipients.

**Objective:**

To assess the incidence of SSI after LT and identify risk factors associated with SSIs and whether SSIs are associated with death and graft loss.

**Design, Setting, and Participants:**

A multicenter cohort study encompassing data on LT performed at all Swiss transplant centers between May 1, 2008, and September 30, 2020, was conducted. Data analyses were performed in 2023.

**Exposure:**

Liver transplant.

**Main Outcomes and Measures:**

Frequency of SSIs within 90 days after transplant, risk factors associated with SSIs, and association of SSIs with 1-year death or graft loss. Surgical site infections were defined according to Centers for Disease Control and Prevention criteria with SSIs occurring within 90 days after LT. For association with posttransplant outcomes, 1-year follow-up data were analyzed.

**Results:**

Among 1333 LT recipients in the Swiss Transplant Cohort Study, 1158 adults were included in analyses. Median age was 57.2 (IQR, 49.3-62.8) years and 792 were men (68.4%). Seventy patients (6.0%) had an SSI. Most SSIs were deep incisional (9 [12.8%]) or organ-space infections (54 [77.1%]). In most SSIs (56 [80.0%]), bacteria were detected, most frequently *Enterococcus* spp (36 of 75 [48.0%]) and *Escherichia coli* (12 of 75 [16.0%]). In multivariable analysis, prior liver transplant (odds ratio [OR] 4.01; 95% CI, 1.44-11.18; *P* = .008) and living liver donation (OR, 4.08; 95% CI, 1.37-12.16; *P* = .01) were independent risk factors associated with SSIs. Surgical site infections were independently associated with graft loss and/or death (hazard ratio [HR], 3.24; 95% CI, 1.82-5.79; *P* < .001); this association was observed in separate analyses on graft loss (HR, 2.97; 95% CI, 1.32-6.68; *P* = .02) and death (HR, 3.25; 95% CI, 1.44-7.35; *P* = .01).

**Conclusions and Relevance:**

The findings of this study suggest that prior liver transplant and living liver donation are independent risk factors associated with SSIs and that SSIs are independently associated with graft loss and/or death, highlighting the relevance of this health care–associated infection.

## Key Points

**Question:**

What is the frequency of surgical site infections (SSIs) after liver transplant?

**Findings:**

In this cohort study including 1158 patients who underwent liver transplant, SSIs were observed in 6.0%. Deep incisional infections contributed to 12.8% of SSIs, organ space infections contributed to 77.1%, and SSIs were independently associated with graft loss and death in the first year after liver transplant.

**Meaning:**

The findings of this study suggest that SSIs are associated with inferior 1-year graft and recipient survival.

## Teaser

This cohort study examines the frequency and outcomes associated with surgical site infections in patients who have undergone liver transplant.

## Introduction

Infections are a major cause of death in the first 180 days after liver transplant (LT).^1^ In Switzerland, the liver is the second most frequently transplanted organ, with approximately 150 transplants performed each year.^2^ In the early posttransplant period, health care–associated infections are among the most common infections in solid organ transplant recipients.^3^ Surgical site infections (SSIs) belong to the most frequent health care–associated infections in point prevalence studies.^4,5^ In nontransplant patients, SSIs have been associated with increased costs, morbidity, and mortality.^6,7^ In a US cohort of liver transplant recipients, SSIs after LT were associated with a higher risk of death or graft loss.^8^ In the literature, the incidence of SSIs after LT varies largely, ranging between 6.6% and 40%.^8,9,10,11^ Data on SSIs after LT derived from multicenter studies are scarce.^10,12^ Published studies are often hampered by different follow-up periods for identifying SSIs and by the frequent use of inconsistent definitions. To address these limitations, we applied Centers for Disease Control and Prevention (CDC) definitions with a slight modification of the follow-up for detection of SSIs, which was extended to 90 days after LT. A recent study supports a longer follow-up period for detection of SSIs, for example, a 2.61-fold increased detection of SSIs has been reported with a follow-up period of at least 60 days.^13^ Schreiber et al^14^ recently analyzed SSIs after kidney transplant and detected several SSIs later than 30 days after transplant.

We analyzed prospectively collected data of the Swiss Transplant Cohort Study (STCS) to assess the incidence of SSIs after LT as the primary objective; identifying risk factors associated with SSI and evaluating whether SSIs are associated with death and graft loss in the first year after LT were secondary objectives.

## Methods

### Design, Population, and Patient-Related Data

This study belonged to a nested project on SSIs after solid organ transplant within the STCS (NCT01204944).^15,16^ For the present study, conducted between May 1, 2008, and September 30, 2020, we analyzed LT recipients aged 18 years or older. For LT recipients with a second LT in the observation period, the first LT in the study period was considered. All Swiss transplant centers (Basel, Bern, Geneva, St Gallen, Lausanne, and Zurich) contribute to the prospective data collection of the STCS. In Switzerland, liver transplants are performed in 3 centers (Bern, Geneva, and Zurich). The STCS is considered highly representative for transplants in Switzerland.^17^ The categorization of SSIs into superficial incisional, deep incisional, and organ-space infections according to CDC criteria^18^ was added retrospectively. The STCS has been approved by the ethics committees of all participating institutions, and this nested study received separate approval. Prior to transplant, patients were requested to grant written informed consent, prompting enrollment into the STCS and inclusion into research projects. No compensation was offered to participants. This study follows the Strengthening the Reporting of Observational Studies in Epidemiology (STROBE) reporting guideline.^19^

### Definition of SSI 

Surgical site infections were defined according to CDC criteria with the modified surveillance period of 90 days after LT. Briefly, SSIs were defined as an infection of the skin, subcutis (ie, superficial incisional), deep soft tissue (ie, deep incisional), or organ space (ie, organ-space infection) within 90 days after the operation, one or more criteria of purulent drainage from the incision, microorganisms cultured from an aseptically obtained specimen or a new drainage or revision), and one or more criteria of pain or tenderness, localized swelling, heat or redness, temperature greater than 38 °C, localized pain or tenderness, and deep abscess.

### Statistical Analysis

Data analysis was conducted from March 1, 2023, to December 16, 2024, with the majority of analysis conducted in 2023. We report baseline recipient characteristics, donor characteristics, and procedure-related and graft-specific variables descriptively. Baseline recipient characteristics that were extracted from the STCS database were sex, age, ethnicity, body mass index, model for end-stage liver disease (MELD) score at LT, Child-Pugh score at LT, source of liver insufficiency, comorbidities, and prior LT. Donor characteristics were sex and age. Procedure-related variables were cold ischemia time, duration of the surgical procedure, induction immunosuppression, maintenance immunosuppression, and transplant center. Graft-specific variables were type of donation and critical liver graft mass (defined as donor liver mass-to-recipient body mass ratio of ≤0.01^8^). Surgical site infections are reported descriptively; variables include incidence rate, time from LT to SSI, SSI category, and causative pathogens. We investigated risk factors associated with SSIs with logistic regression. Variables considered in the multivariable analysis were chosen based on a combination of published evidence (ie, duration of surgery,^8^ cold ischemia time,^20^ critical liver graft mass,^8^ and prior liver transplant^21^), the results of univariable logistic regression combined with biological or clinical plausibility (ie, type of donation) and basic epidemiologic variables (ie, recipient age and sex). For the multivariable analyses, we ran a model with multiple imputation of missing variables using the package mice in R, version 4.2.1 (R Foundation for Statistical Computing).^22^ As sensitivity analyses, we performed a multivariable analysis considering only cases with complete information for all addressed variables and a multivariable analysis focusing on the subset of deep incisional and organ-space infections, which also included the transplant center as a variable.

Transplant outcomes, encompassing death and graft loss, were extracted from the STCS dataset and presented for a 1-year follow-up. Cumulative incidences for death and graft loss as competing risks were calculated. To investigate associations of SSIs and transplant outcomes, we performed cause-specific Cox proportional hazards models with SSI as a time-dependent variable; the exposure to SSIs was coded as permanent exposure. Cause-specific Cox proportional hazards models were fit for the combined end point of death and/or graft loss correcting for known predictors,^8,20,23,24,25^ presence and absence of SSIs, the results of univariable regression combined with biological or clinical plausibility, and basic epidemiologic variables (ie, recipient age and sex). To ensure that the estimated association between SSI and the combined end point was robust, we also considered an analogous model where we allowed nonlinear effects for the numerical variables recipient age, donor age, and MELD score via restricted cubic splines with 4 knots, using the package rms in R.^26^ Additionally, we performed cause-specific Cox proportional hazards models for the end points death and graft loss separately. A threshold value of *P* < .05 was considered statistically significant. R, version 4.2.1 R Foundation for Statistical Computing), was used for statistical analysis and visualization.^27^ Data analyses were performed in 2023 to enable use of STCS data that had already undergone in-depth quality checks.

## Results

### Study Population

**Figure 1.**
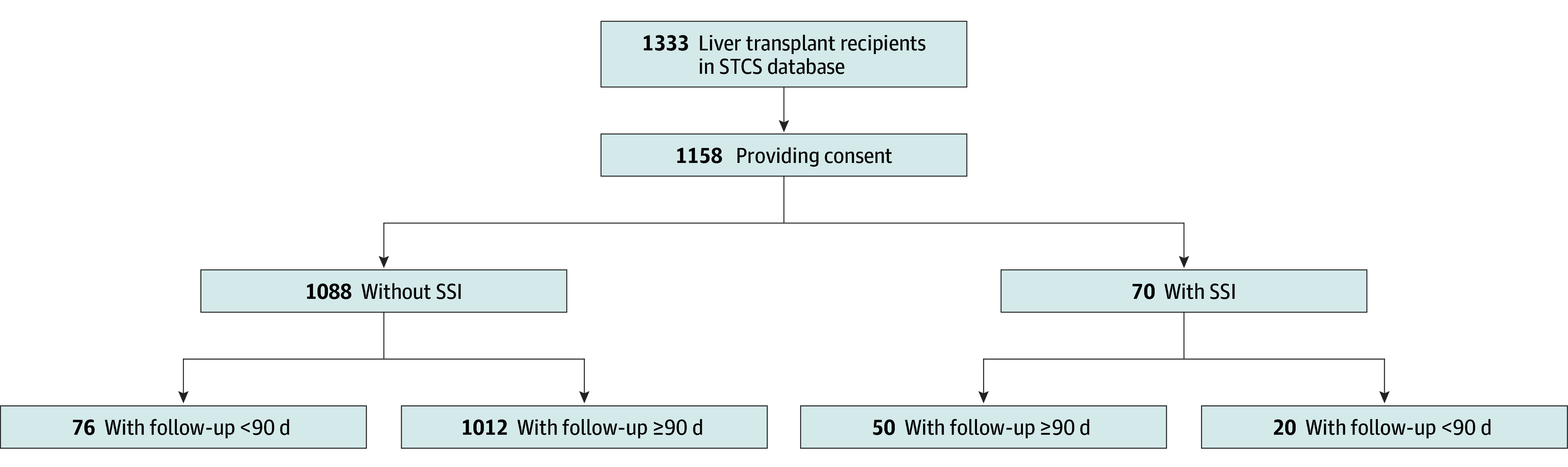
Study Population Selection SSI indicates surgical site infection; STCS, Swiss Transplant Cohort Study.

The study included a total of 1158 LT recipients with a median age of 57.2 (IQR, 49.3-62.8) years (Table 1). The study population selection is depicted in Figure 1. More LT recipients were male (792 [68.4%] vs 366 [31.6%]). A total of 1064 (91.9%) LT recipients were White. Median body mass index (calculated as weight in kilograms divided by height in meters squared) of LT recipients was 25.6 (95% CI, 22.8-29.4).Almost all LT recipients (1117 [96.5%]) were included after their first transplant. The most frequent reasons for liver transplant were hepatocellular carcinoma (258 [22.3%]), alcoholic liver cirrhosis (230 [19.9%]), and hepatitis C (185 [16.0%]). Most liver grafts were derived from deceased donors (1095 [94.6%]) with a predominance of donation after brain death (951 [82.1%]). Induction immunosuppression predominantly consisted of basiliximab (930 [80.3%], 9 cases with coadministration of antithymocyte globulin). Maintenance immunosuppression contained tacrolimus in most patients (1004 [86.7%]). All transplant centers had infection prevention and control guidelines for SSIs prevention in place. Common elements included surgical hand disinfection, skin disinfection, sterile draping, aseptic technique, administration of antibiotic prophylaxis within 30 to 60 minutes before incision, and maintenance of normothermia during the perioperative period. Routine perioperative antibiotic prophylaxis was either amoxicillin with clavulanate (1 center), cefuroxime (1 center), or piperacillin with tazobactam (1 center) administered within 30 to 60 minutes before incision.

**Table 1.** Baseline Characteristics of 1158 Liver Transplant Recipients

Variable	No. (%)		
	Overall (N = 1158)	No SSI (n = 1088)	SSI (n = 70)
Recipient sex			
Female	366 (31.6)	339 (31.2)	27 (38.6)
Male	792 (68.4)	749 (68.8)	43 (61.4)
Age, median (IQR), y	57.2 (49.3-62.8)	57.1 (49.4-62.7)	57.7 (49.2-63.3)
Ethnicity^a^			
African	37 (3.2)	35 (3.2)	2 (2.9)
Asian	32 (2.7)	29 (2.7)	3 (4.3)
White	1064 (91.9)	1001 (92.0)	63 (90.0)
Other	16 (1.4)	14 (1.2)	2 (2.9)
BMI, median (IQR)^b^	25.6 (22.8-29.4)	25.7 (22.8-29.4)	24.7 (22.6-29.2)
MELD score at transplant, median (IQR)^c^	14 (9-24)	14 (9-23)	15 (10-25)
Child-Pugh score at transplant, median (IQR)^d^	8 (6-10)	8 (6-10)	8 (6-10)
Etiology			
Hepatocellular carcinoma	258 (22.3)	246 (22.6)	12 (17.1)
Alcoholic liver cirrhosis	230 (19.9)	222 (20.4)	8 (11.4)
Hepatitis C	185 (16.0)	176 (16.2)	9 (12.9)
Hepatitis B	89 (7.7)	81 (7.4)	8 (11.4)
Primary biliary cirrhosis	41 (3.5)	35 (3.2)	6 (8.6)
Primary sclerosing cholangitis	41 (3.5)	37 (3.4)	4 (5.7)
Cholangiocarcinoma	22 (1.9)	16 (1.5)	6 (8.6)
Drug-associated	19 (1.6)	19 (1.7)	0 (0.0%
Autoimmune hepatitis	18 (1.6)	17 (1.6)	1 (1.4)
Secondary biliary cirrhosis	17 (1.5)	14 (1.3)	3 (4.3)
Morbus Wilson	15 (1.3)	15 (1.4)	0
Previous graft failure	10 (0.9)	8 (0.7)	2 (2.9)
Other reasons	213 (18.4)	202 (18.6)	11 (15.7)
Comorbidities			
Diabetes at transplant	283 (24.4)	273 (25.1)	10 (14.3)
Cerebrovascular disease	34 (2.9)	33 (3.0)	1 (1.4)
Coronary artery disease	124 (10.7)	116 (10.7)	8 (11.4)
Peripheral artery disease	55 (4.8)	52 (4.8)	3 (4.3)
Prior liver transplant	35 (3.0)	28 (2.6)	7 (10.0)
Cold ischemia time, median (IQR), min^e^	391.0 (318.3-477.7)	393.0 (320.0-480.0)	371.5 (255-454.8)
Duration of surgical procedure, median (IQR), h^f^	6.00 (4.47-8.17)	6.00 (4.47-8.17)	6.00 (4.54-8.20)
Induction immunosuppression^g^			
Antithymocyte globulin	19 (1.64)	19 (1.74)	0
Basiliximab	930 (80.3)	874 (80.3)	56 (80.0)
Other	209 (18.0)	195 (17.9)	14 (20.0)
Maintenance immunosuppression^h^			
Tacrolimus-containing regimen	1004 (86.7)	947 (87.0)	57 (81.4)
Cyclosporin-containing regimen	75 (6.5)	70 (6.4)	5 (7.1)
Other	66 (5.7)	71 (6.5)	6 (8.5)
Donor sex (male)^i^	654 (56.6)	617 (56.8)	37 (52.9)
Donor age at donation, median (IQR), y^j^	56.0 (42.0-68.0)	56.0 (43.0-68.0)	52.0 (37.0-64.2)
Type of donation			
DBD	951 (82.1)	899 (82.6)	52 (74.3)
DCD	144 (12.4)	138 (12.7)	6 (8.6)
Living	63 (5.4)	51 (4.7)	12 (17.1)
Critical liver graft mass^k^	40 (4.6)	32 (4.0)	8 (14.5)

Abbreviations: BMI, body mass index (calculated as weight in kilograms divided by height in meters squared); DBD, donation after brain death; DCD, donation after cardiocirculatory death; MELD, model for end-stage liver disease; SSI, surgical site infection.

^a^ Ethnicity missing for 9 (0.78%) liver transplant recipients.

^b^ BMI missing for 11 (0.95%) liver transplant recipients.

^c^ MELD score missing for 24 (2.07%) liver transplant recipients.

^d^ Child-Pugh score missing for 30 (2.59%) liver transplant recipients.

^e^ Cold ischemia time missing for 64 (5.53%) liver transplant recipients.

^f^ Duration of surgical procedure missing for 33 (2.85%) liver transplant recipients.

^g^ Nine (0.8%) liver transplant recipients received both antithymocyte globulin and basiliximab.

^h^ Maintenance immunosuppressive regimen started within the first 2 weeks after transplant; data for 13 (1.1%) patients unknown.

^i^ Donor sex missing for 2 (0.17%) liver transplant recipients.

^j^ Donor age missing for 12 (1.03%) liver transplant recipients.

^k^ Defined as donor liver mass-to-recipient body mass ratio of 0.01 or less. Data for 295 (25.5%) missing.

### Incidence and Causative Pathogens of SSIs

**Figure 2.**
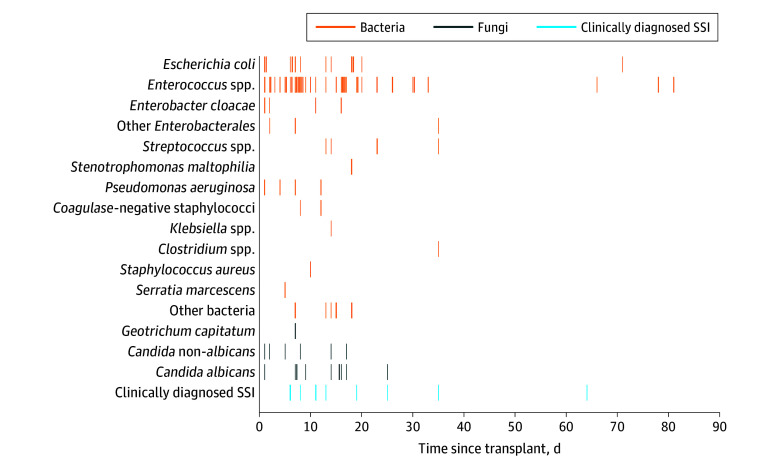
Temporal Distribution of Detected Pathogens in Surgical Site Infections (SSIs) After Liver Transplant All *Escherichia coli* isolates, 3 *Enterobacter cloacae* isolates, 1 *Klebsiella* spp isolate, and 1 *Serratia marcescens* isolate were extended-spectrum β-lactamase producers. None of the *Staphylococcus aureus* isolates was methicillin-resistant and none of the *Enterococcus* spp was vancomycin-resistant. In clinically diagnosed SSI, no causative pathogens were detected.

Of 1158 LT recipients, 70 individuals (6.0%) developed an SSI within 90 days after transplant (Figure 2). The median time from LT to SSI was 13 (IQR, 7-19) days. Superficial incisional infections contributed to 7 (10.0%), deep incisional infections to 9 (12.8%), and organ-space infections to 54 (77.1%) SSIs. Sixteen SSIs (22.9%) were polymicrobial. In most SSIs, bacteria were identified (56 [80.0%]). Among all 75 detected bacteria, the most frequently identified were *Enterococcus* spp (36 of 75 [48.0%]), *Escherichia coli* (12 of 75 [16.0%]), *Streptococcus* spp (4 of 75 [5.3%]), *Enterobacter cloacae* (4 of 75 [5.3%]), and *Pseudomonas aeruginosa* (4 of 75 [5.3%]). Among a total of 16 detected fungal pathogens, yeasts predominated (15 of 16 [93.8%]). *Candida albicans* was found in 9 of 16 (56.3%) SSIs, *Candida* non-*albicans* in 6 of 16 (37.5%) SSIs, and *Geotrichum capitatum* in 1 of 16 (6.3%) SSIs. In 21 LT recipients (30.0%) with SSIs, revision surgery was performed. Biliary complications were reported in 32 LT recipients (45.7%) with SSIs.

### Risk Factors Associated With SSI After LT

In univariable analyses, a ratio of graft organ weight to recipient weight less than or equal to 0.01 (odds ratio [OR], 4.73; 95% CI, 1.93-10.50; *P* < .001), prior liver transplant (OR, 3.21; 95% CI, 1.06-7.97; *P* = .02), and living liver donation (OR, 4.32; 95% CI, 2.03-8.52; *P* < .001) were risk factors (Table 2). In multivariable analyses, a prior liver transplant (OR, 4.01; 95% CI, 1.44-11.18; *P* = .008) and living liver donation (OR, 4.08; 95% CI, 1.37-12.16; *P* = .01) were independently associated with SSIs. In a sensitivity analysis focusing exclusively on complete datasets, the multivariable analyses found an independent association of the variable prior liver transplant (OR, 5.20; 95% CI, 1.39-15.81; *P* = .007) but the finding for living liver donation (OR, 4.18; 95% CI, 0.93-17.65; *P* = .06) was not statistically significant (eTable 1 in Supplement 1). With a further sensitivity analysis focusing on the subset of deep incisional and organ-space infections, prior liver transplant (OR, 4.72; 95% CI, 1.23-14.86; *P* = .01) was associated with SSIs (eTable 2 in Supplement 1).

**Table 2.** Risk Factors Associated With Surgical Site Infections Within 90 Days After Liver Transplant

Characteristic	Univariable		Multivariable^a^	
	OR (95% CI)	*P* value	OR (95% CI)	*P* value
Recipient sex (male)	0.80 (0.47-1.37)	.40	0.79 (0.45-1.39)	.42
Recipient age (per 10-y increase)	1.08 (0.87-1.36)	.53	1.16 (0.92-1.47)	.21
BMI	0.99 (0.94-1.04)	.62	NA	NA
Preexisting diabetes mellitus	0.56 (0.27-1.07)	.10	NA	NA
Prior liver transplant	3.21 (1.06-7.97)	.02	4.01 (1.44-11.18)	.008
Presence of ascites at LT	1.19 (0.63-2.26)	.59	NA	NA
MELD score at LT	1.01 (0.98-1.03)	.47	NA	NA
Child-Pugh score at LT	1.03 (0.93-1.13)	.63	NA	NA
Cold ischemia time (per 10 min)	0.99 (0.97-1.01)	.15	1.01 (0.99-1.03)	.56
Duration of transplant surgery (per h)	1.04 (0.95-1.13)	.43	0.97 (0.88-1.08)	.60
Type of donation (living)	4.32 (2.03-8.52)	<.001	4.08 (1.37-12.16)	.01
Critical liver graft mass (donor liver mass-to-recipient body mass ≤0.01)	4.73 (1.93-10.50)	<.001	2.24 (0.88-5.65)	.09
Donor sex (male)	0.72 (0.43-1.20)	.20	NA	NA
Donor age (per 10-y increase)	1.00 (0.97-1.07)	.81	NA	NA
Induction immunosuppression			NA	NA
Basiliximab	1 [Reference]	.85	NA	NA
Antithymocyte globulin or other induction therapy	0.94 (0.47-1.17)		NA	
Maintenance regimen			NA	NA
Tacrolimus containing	1 [Reference]	.38	NA	NA
Cyclosporine containing or other	1.37 (0.64-2.65)		NA	

Abbreviations: BMI, body mass index; LT, liver transplant; OR, odds ratio; MELD, model for end-stage liver disease; NA, not applicable.

^a^ Multivariable analyses with imputation of missing variables.

### Associations of SSIs and Posttransplant Outcomes

Crude outcome data on a follow-up of 1 year are summarized in eTable 3 in Supplement 1. In univariable cause-specific Cox proportional hazards models, associations of the combined outcome death/graft loss and an increase in MELD score at the time of LT (per-point increased hazard ratio [HR], 1.02; 95% CI, 1.00-1.03; *P* = .02), living liver donation (HR, 2.43; 95% CI, 1.49-3.97; *P* < .001), induction therapy other than basiliximab (HR, 1.54; 95% CI, 1.09-2.18; *P* = .01), and SSIs (HR, 3.20; 95% CI, 1.83-5.62; *P* < .001) were detected (Table 3).

**Table 3.** Cause-Specific Cox Proportional Hazards Model for Risk of Death and/or Graft Loss in the First Year After Liver Transplantation Treating Surgical Site Infection as Time Dependent

Characteristic	Univariable		Multivariable	
	HR (95% CI)	*P* value	HR (95% CI)	*P* value
Recipient sex (male)	0.89 (0.64-1.24)	.48	0.96 (0.66-1.41)	.85
Recipient age (per 10-y increase)	1.01 (0.88-1.16)	.86	1.10 (0.93-1.29)	.26
MELD score	1.02 (1.00-1.03)	.02	1.02 (1.00-1.04)	.02
Induction immunosuppression				.
Basiliximab	1 [Reference]	.01	1 [Reference]	07
Antithymocyte globulin or other induction therapy	1.54 (1.09-2.18)		1.45 (0.98-2.16)	
Maintenance regimen				
Tacrolimus containing	1 [Reference]	<.001	1 [Reference]	<.001
Cyclosporine containing or other	2.95 (2.05-4.23)		3.58 (2.43-5.26)	
Type of donation (living)	2.43 (1.49-3.97)	<.001	5.09 (2.38-10.87)	<.001
Donor age (per 10-y increase)	1.05 (0.96-1.15)	.29	1.13 (1.02-1.25)	.02
Cold ischemia time (per 10 min)	1.00 (0.99-1.01)	.75	1.02 (1.00-1.03)	.02
Duration of transplant surgery (per h)	0.99 (0.93-1.05)	.61	0.96 (0.90-1.03)	.27
SSI	3.20 (1.83-5.62)	<.001	3.24 (1.82-5.79)	<.001

Abbreviations: HR, hazard ratio; MELD, model for end-stage liver disease; SSI, surgical site infection.

In multivariable analyses, SSI (HR, 3.24; 95% CI, 1.82-5.79; *P* < .001), an increase in the MELD score at the time of LT (per-point increased HR, 1.02; 95% CI, 1.00-1.04; *P* = .02), living liver donation (HR, 5.09; 95% CI, 2.38-10.87; *P* < .001), higher donor age (per 10-year increased HR, 1.13; 95% CI, 1.02-1.25; *P* = .02), longer cold ischemia time (per 10-minute increased HR, 1.02; 95% CI, 1.00-1.03; *P* = .02), and maintenance immunosuppression other than tacrolimus (HR 3.58; 95% CI, 2.43-5.26; *P* < .001) were independently associated with death and/or graft loss. Induction immunosuppression other than basiliximab was not associated with death and/or graft loss (HR, 1.45; 95% CI, 0.98-2.16; *P* = .07) (Table 3).

Univariable and multivariable cause-specific Cox proportional hazards models for the outcomes death and graft loss separately are provided in eTable 4 and eTable 5 in Supplement 1. These analyses supported an independent association of SSI and death (HR, 3.25; 95% CI, 1.44-7.35; *P* = .01) as well as SSI and graft loss (HR, 2.97; 95% CI, 1.32-6.68; *P* = .02).

In a sensitivity analysis, allowing nonlinearities for continuous variables via restricted cubic splines, the multivariable analysis found an independent association between SSI (HR 3.30, 95% CI 1.85-5.89, *P* < .001) and death/graft loss (eTable 6, eFigure in Supplement 1).

## Discussion

Of 1158 LT recipients, 70 individuals (6.0%) developed an SSI. In 80.0% of SSIs, bacteria were detected, most frequently *Enterococcus* spp and *Escherichia coli*. Prior liver transplant and living donor were independently associated with SSIs. Surgical site infections were independently associated with increased hazards for death and/or graft loss in the first year after liver transplant.

In the present study, we report an SSI rate of 6.0% after LT. Most published studies reported higher SSI rates after LT, frequently around 20% or even higher.^8,9,11,20,28,29^ Asensio et al^10^ reported more comparable SSI rates in a large study with inclusion of 1222 LT recipients. Our SSI rate is similar to rates recently reported for open abdominal surgery in general.^30,31^ In the general patient population, SSIs have been associated with prolonged hospital stay, excess costs, and even mortality for some populations.^32^ For many aspects, such as morbidity due to operative revisions and antibiotic therapy, the burden of disease associated with SSIs might be similar for LT recipients, but after LT there is also the risk of graft loss and need for retransplant.

In the present study, organ-space and deep incisional infections predominated. The large proportion of more extensive SSI is in line with earlier studies.^10,11,20,28^ In contrast, Hellinger et al^8^ reported a majority of superficial incisional SSIs.

Approximately one-fourth of all SSIs were caused by multiple pathogens. Polymicrobial infections are common in SSIs after liver transplant.^9,11,20^ Among the causative bacteria, we most frequently found *Enterococcus* spp and *E coli*. Similar to our findings, García Prado et al^11^ and Asensio et al^10^ reported these bacteria as the most common in their studies on SSI after liver transplant. In our study, none of the *Staphylococcus aureus* isolates was methicillin-resistant and none of the *Enterococcus* spp isolates was vancomycin-resistant. In an official report on antibiotic resistance published in 2022, Switzerland had a methicillin-resistant *S aureus* rate below 5%.^33^ In 2020, vancomycin-resistance of *Enterococcus* spp was still rare in Switzerland, around 2.9%.^33^ Fungi were detected in 12 SSIs; among these, fungal pathogens *Candida* spp were most common, with a predominance of *C albicans*. Prior studies support the relevance of *Candida* spp as causative pathogens after liver transplant.^8,9,20,28^ Asensio et al^10^ also detected *C albicans* more often than non-*albicans Candida* spp, whereas Freire et al^20^ found a predominance of non-*albicans Candida* spp

Independent risk factors associated with the development of an SSI were prior liver transplant and living donor. Liver retransplant has been described as a risk factor for SSI by Asensio et al^10^ and Freire et a.l^20^ A study comparing infections in first liver transplant recipients and liver retransplant recipients also found more SSIs after liver retransplant.^21^ The increased risk after liver retransplant could be explained by a more complex intervention, for example, due to adhesions after antecedent liver surgery or prolonged prior immunosuppressive treatment. In living liver donation, split livers are used as grafts, resulting in a large wound surface that might favor an SSI. Data on SSIs after living liver donation are scarce. Iinuma et al^34^ reported on a cohort of living donor liver transplant recipients an SSI rate of around 40%; the authors interpreted this finding as comparable to contemporary reports on SSIs after cadaveric liver transplant. Similarly, Yamamoto et al^35^ reported an SSI rate among adult recipients of living donor transplants as 30.3% and 41.3% for 2 different periods of time and interpreted these rates as higher than those after cadaveric liver transplant. The root cause of SSIs in our study remains speculative. Considering the large proportion of biliary complications among LT recipients with SSIs, contamination originating from the biliary system might have caused subsequent SSIs.

In the present study, all transplant centers had infection prevention and control guidelines for SSI prevention implemented. Current guidelines recommend as essential practices several additional elements,^36^ such as control of blood glucose levels during the immediate postoperative period. As all LT recipients are hospitalized in an intensive care unit immediately after the transplant procedure, regular glucose control and corrective measures can be assumed. In addition, monitoring of process measures and education of surgeons and perioperative personnel have been recommended. A decrease in SSI rates seems achievable. A systematic review and meta-analysis suggested a preventable proportion of SSIs of around 50%.^37^

In our analyses for posttransplant outcomes, we found an independent association of SSIs and the combined end point death and/or graft loss in the first year post transplant. This association was also detected if we analyzed the outcomes graft loss or death separately. Similar to our findings, Hellinger et al^8^ reported an association between SSIs and the combined end point graft loss or death and the end point graft loss in a follow-up period of 1 year after LT. Reid et al^9^ also found in their study on intraabdominal infections soon after LT an association between intraabdominal infections and graft failure, but they did not identify an association with death. García Prado et al^11^ reported a prolonged hospital stay in patients with SSIs after liver transplant but did not identify an association with death and did not investigate graft losses.

Our analysis regarding the outcome graft loss and/or death focused on associations with SSIs. Hence, the interpretation of other variables that might also influence the incidence of SSI should be done with some caution. Higher donor age was also independently associated with the combined end point of death and/or graft loss in the first year post transplant. In our study, the association of the variable donor age and 1-year outcomes was due to the association with graft loss. A systematic review and meta-analysis reported that cadaveric grafts from older donors were associated with both graft failure and mortality.^38^

### Strengths and Limitations

Strengths of the present study are the multicenter design, the extended surveillance period of 90 days after LT ensuring comprehensive detection of SSIs, the prolonged period of standardized data collection, the use of predominantly prospectively collected data, and the use of well-established uniform CDC SSI definitions. The robustness of our findings regarding associations of SSI and posttransplant outcomes was supported by extensive sensitivity analyses.

Our study also has some limitations. Information on the individually administered perioperative antibiotic prophylaxis and on normothermia during the surgical procedure was missing. Our dataset also did not include information on the surgeon, surgeon’s experience, surgical techniques (eg, type of anastomoses), American Society of Anesthesiologists score of the recipient, postoperative glucose levels, or the number of red blood cell transfusions; thus, we could not adjust for these variables in a risk factor analysis. Similarly, our dataset did not include information on the type of ward in which the patient was hospitalized prior to LT and on the urgency of LT. The predominance of White individuals in our cohort and a median body mass index at the border of healthy weight to overweight might limit generalizability of our findings for other regions.^39^ Our analyses on secondary outcomes were associative and should be interpreted accordingly.

## Conclusions

In this cohort study, SSIs were observed in 6.0% of LT recipients and were independently associated with graft loss and death. Future efforts are indicated to prevent SSIs in this vulnerable population.

## Notes

Supplement 1. **eTable 1.** Sensitivity Analysis of Risk Factors for Surgical Site Infections Within 90 Days After Liver Transplantation (Multivariable Model Restricted to Complete Cases) **eTable 2.** Sensitivity Analysis of Risk Factors for the Subset of Deep Incisional and Organ/Space Infections Within 90 Days After Liver Transplantation (Multivariable Model Restricted to Complete Cases) **eTable 3.** Post-Transplant Outcomes During 1-Year Follow-Up After Liver Transplantation **eTable 4.** Cause-Specific Cox Proportional Hazard Model for Risk Of Death in the First Year After Liver Transplantation Treating Surgical Site Infection as Time-Dependent **eTable 5.** Cause-Specific Cox Proportional Hazard Model For Risk of Graft Loss in the First Year After Liver Transplantation Treating Surgical Site Infection as Time-Dependent **eTable 6.** Sensitivity Analysis for Cause-Specific Cox Proportional Hazard Models for Risk of Death and/or Graft Loss in the First Year After Liver Transplantation With Nonlinear Effects for Numerical Variables via Restricted Cubic Splines Treating Surgical Site Infection as Time-Dependent **eFigure.** Visualization of Recipient Age/MELD/Donor Age-Hazard Ratio Relationships From Cause-Specific Cox Proportional Hazards Models for Risk of Death and/or Graft Loss in the First Year After Liver Transplantation

Supplement 2. **Nonauthor Collaborators.** The Swiss Transplant Cohort Study

Supplement 3.
Data Sharing Statement
