# Supplementary material for: Surgical Site Infections, Risk Factors, and Outcomes After Liver Transplant
Source: JAMA Netw Open. 2025 Mar 21;8(3):e251333. doi: 10.1001/jamanetworkopen.2025.1333 (PMC11929024; doi:10.1001/jamanetworkopen.2025.1333)
Supplement: Supplement 1. — eTable 1. Sensitivity Analysis of Risk Factors for Surgical Site Infections Within 90 Days After Liver Transplantation (Multivariable Model Restricted to Complete Cases) eTable 2. Sensitivity Analysis of Risk Factors for the Subset of Deep Incisional and Organ/Space Infections Within 90 Days After Liver Transplantation (Multivariable Model Restricted to Complete Cases) eTable 3. Post-Transplant Outcomes During 1-Year Follow-Up After Liver Transplantation eTable 4. Cause-Specific Cox Proportional Hazard Model for Risk Of Death in the First Year After Liver Transplantation Treating Surgical Site Infection as Time-Dependent eTable 5. Cause-Specific Cox Proportional Hazard Model For Risk of Graft Loss in the First Year After Liver Transplantation Treating Surgical Site Infection as Time-Dependent eTable 6. Sensitivity Analysis for Cause-Specific Cox Proportional Hazard Models for Risk of Death and/or Graft Loss in the First Year After Liver Transplantation With Nonlinear Effects for Numerical Variables via Restricted Cubic Splines Treating Surgical Site Infection as Time-Dependent eFigure. Visualization of Recipient Age/MELD/Donor Age-Hazard Ratio Relationships From Cause-Specific Cox Proportional Hazards Models for Risk of Death and/or Graft Loss in the First Year After Liver Transplantation [file jamanetwopen-e251333-s001.pdf]

Supplementary Online Content

Schreiber PW, Hoessly LD, Boggian K, et al; the Swiss Transplant Cohort Study. Surgical site infections, risk factors, and outcomes after liver transplant. *JAMA Netw Open*. 2025;8(3):e251333. doi:10.1001/jamanetworkopen.2025.1333

eTable 1. Sensitivity Analysis of Risk Factors for Surgical Site Infections Within 90 Days After Liver Transplantation (Multivariable Model Restricted to Complete Cases)

eTable 2. Sensitivity Analysis of Risk Factors for the Subset of Deep Incisional and Organ/Space Infections Within 90 Days After Liver Transplantation (Multivariable Model Restricted to Complete Cases)

eTable 3. Post-Transplant Outcomes During 1-Year Follow-Up After Liver Transplantation

eTable 4. Cause-Specific Cox Proportional Hazard Model for Risk Of Death in the First Year After Liver Transplantation Treating Surgical Site Infection as Time-Dependent

eTable 5. Cause-Specific Cox Proportional Hazard Model For Risk Of Graft Loss in the First Year After Liver Transplantation Treating Surgical Site Infection as Time-Dependent

eTable 6. Sensitivity Analysis for Cause-Specific Cox Proportional Hazard Models for Risk of Death and/or Graft Loss in the First Year After Liver Transplantation With Nonlinear Effects for Numerical Variables via Restricted Cubic Splines Treating Surgical Site Infection as Time-Dependent

eFigure. Visualization of Recipient Age/MELD/Donor Age-Hazard Ratio Relationships From Cause-Specific Cox Proportional Hazards Models for Risk of Death and/or Graft Loss in the First Year After Liver Transplantation

This supplementary material has been provided by the authors to give readers additional information about their work.

© 2025 Schreiber PW et al.

eTable 1: Sensitivity analysis of risk factors for surgical site infections within 90 days after liver transplantation (multivariable model restricted to complete cases)

	**univariable**		**multivariable***	
**Characteristic**	**OR (95% CI)**	**p**	**OR (95% CI)**	**p**
Recipient sex (male)	0.80 (0.47, 1.37)	0.40	0.89 (0.47, 1.75)	0.73
Recipient age (per ten year increase)	1.07 (0.87, 1.36)	0.54	1.22 (0.93, 1.63)	0.16
BMI	0.99 (0.94, 1.04)	0.62		
Preexisting diabetes mellitus	0.56 (0.27, 1.07)	0.10		
Prior liver transplant	3.21 (1.06, 7.97)	0.02	5.20 (1.39, 15.81)	0.007
Presence of ascites at L-Tx	1.19 (0.63, 2.27)	0.58		
MELD score at L-Tx	1.01 (0.98, 1.03)	0.47		
Child-Pugh score at L-Tx	1.03 (0.93, 1.13)	0.61		
Cold ischemia time (per ten minutes)	0.99 (0.97, 1.01)	0.15	1.01 (0.98, 1.03)	0.61
Duration of transplant surgery (per hour)	1.04 (0.95, 1.13)	0.41	0.98 (0.87, 1.10)	0.77
Type of donation (living)	4.32 (2.03, 8.52)	<0.001	4.18 (0.93, 17.65)	0.06
Critical liver graft mass (donor liver mass-to-recipient body mass ≤ 0.01)	4.73 (1.93, 10.50)	<0.001	2.70 (0.82, 7.89)	0.09
Donor sex (male)	0.72 (0.43, 1.20)	0.20		
Donor age (per ten year increase)	0.89 (0.78, 1.03)	0.11		
Induction immunosuppression		0.72		
°°°Basiliximab	Reference			
°°°Antithymocyte globulin (ATG) or other induction therapy	0.89 (0.45, 1.64)			
Maintenance regimen		0.38		
°°°Tacrolimus-containing	Reference			
°°°Cyclosporin A-containing or other	1.37 (0.64, 2.64)			

Abbreviations: ATG antithymocyte globulin, BMI body mass index, OR odds ratio, 95% CI 95% confidence interval, MELD model for end-stage liver disease

* Multivariable analyses restricted to complete cases

© 2025 Schreiber PW et al.

eTable 2: Sensitivity analysis of risk factors for the subset of deep incisional and organ/space infections within 90 days after liver transplantation (multivariable model restricted to complete cases)

	univariable		multivariable*	
**Characteristic**	**OR (95% CI)**	**p**	**OR (95% CI)**	**p**
Recipient sex (male)	0.68 (0.40, 1.15)	0.14	0.69 (0.36, 1.35)	0.27
Recipient age (per ten year increase)	1.10 (0.88, 1.39)	0.43	1.18 (0.90, 1.58)	0.25
BMI	0.98 (0.93, 1.04)	0.52		
Preexisting diabetes mellitus	0.44 (0.19, 0.88)	0.03		
Prior liver transplant	3.27 (1.08, 8.12)	0.02	4.72 (1.23, 14.86)	0.01
Presence of ascites at L-Tx	1.33 (0.70, 2.54)	0.38		
MELD score at L-Tx	1.01 (0.99, 1.04)	0.42		
Child-Pugh score at L-Tx	1.04 (0.94, 1.15)	0.41		
Cold ischemia time (per ten minutes)	0.99 (0.97, 1.01)	0.23	1.02 (0.99, 1.05)	0.17
Duration of transplant surgery (per hour)	1.06 (0.97, 1.16)	0.16	1.15 (1.01, 1.32)	0.04
Type of donation (living)	5.01 (2.42, 9.75)	<0.001	3.80 (0.70, 19.09)	0.11
Critical liver graft mass (donor liver mass-to-recipient body mass ≤ 0.01)	4.85 (1.98, 10.79)	<0.001	2.61 (0.77, 8.13)	0.11
Donor sex (male)	0.80 (0.48, 1.33)	0.38		
Donor age (per ten year increase)	0.88 (0.76, 1.00)	0.05		
Induction immunosuppression		0.85		
°°°Basiliximab	Reference			
°°°Antithymocyte globulin (ATG) or other induction therapy	1.06 (0.55, 1.94)			
Maintenance regimen		0.19		
°°°Tacrolimus-containing	Reference			
°°°Cyclosporin A-containing or other	1.58 (0.76, 2.99)			
Center				
°°°A	Reference		Reference	
°°°B	0.30 (0.13, 0.63)	0.003	0.10 (0.03, 0.34)	<0.001
°°°C	0.81 (0.46, 1.44)	0.46	0.42 (0.20, 0.89)	0.02

Abbreviations: ATG antithymocyte globulin, BMI body mass index, OR odds ratio, 95% CI 95% confidence interval, MELD model for end-stage liver disease

* Multivariable analyses restricted to complete cases

© 2025 Schreiber PW et al.

eTable 3: Post-transplant outcomes during 1-year follow-up after liver transplantation

	Liver transplantation[a] (N = 1158)			
	SSI (N = 70)		No SSI (N = 1088)	
**Death**				
Time period after transplantation	first 90d	after 90d	first 90d	after 90d
Number of patients, (N, %)	13 (8.57)	3 (4.29)	55 (5.06)	41 (3.77)
Time to event, days, (median, [IQR])	68.0 [39.5, 104.5]		68.0 [11.75, 214.75]	
**Graft loss**				
Time period after transplantation	first 90d	after 90d	first 90d	after 90d
Number of patients, (N, %)	16 (22.86)	1 (1.43)	48 (4.41)	30 (2.76)
Time to event, days, (median, [IQR])	12.0 [1.0, 36.0]		18.0 [0.0,149.8]	

[a] Four (0.3%) patients were lost to follow-up in the first year after liver transplantation.

© 2025 Schreiber PW et al.

eTable 4: Cause-specific Cox proportional hazard model for risk of death in the first year after liver transplantation treating surgical site infection as time-dependent

	Death			
	univariable		multivariable	
**Characteristic**	**HR (95%CI)**	**p**	**HR (95%CI)**	**p**
Recipient sex (male)	0.82 (0.49, 1.38)	0.45	0.92 (0.51, 1.65)	0.78
Recipient age (per ten year increase)	1.15 (0.88, 1.41)	0.36	1.36 (1.02, 1.81)	0.03
MELD score	1.05 (1.03, 1.08)	<0.001	1.06 (1.03, 1.09)	<0.001
Induction immunosuppression		0.31		
°°°Basiliximab	Reference			
°°°Antithymocyte globulin (ATG) or other induction therapy	1.34 (0.76, 2.37)			
Maintenance regimen		<0.001		<0.001
°°°Tacrolimus-containing	Reference		Reference	
°°°Cyclosporin A-containing or other	3.53 (2.00, 6.22)		4.61 (2.53, 8.40)	
Type of donation (living)	2.05 (0.88, 4.76)	0.09	3.76 (1.35, 10.46)	0.02
Donor age (per ten year increase)	1.01 (0.87, 1.17)	0.94	1.05 (0.89, 1.24)	0.56
Cold ischemia time (per ten minutes)	1.00 (0.98, 1.02)	0.78		
Duration of transplant surgery (per hour)	0.95 (0.86, 1.06)	0.36		
SSI	3.21 (1.45, 7.11)	0.004	3.25 (1.44, 7.35)	0.01

Abbreviations: HR hazard ratio, 95% CI 95% confidence interval, MELD model for end-stage liver disease

© 2025 Schreiber PW et al.

eTable 5: Cause-specific Cox proportional hazard model for risk of graft loss in the first year after liver transplantation treating surgical site infection as time-dependent

	**Graft loss**			
	**univariable**		**multivariable**	
**Characteristic**	**HR (95%CI)**	**p**	**HR (95%CI)**	**p**
Recipient sex (male)	0.94 (0.61, 1.44)	0.76	0.99 (0.62, 1.60)	0.99
Recipient age (per ten year increase)	0.96 (0.80, 1.14)	0.61	0.99 (0.82, 1.20)	0.92
MELD score	1.00 (0.98, 1.02)	0.83		
Induction immunosuppression		0.02		0.13
°°°Basiliximab	Reference		Reference	
°°°Antithymocyte globulin (ATG) or other induction therapy	1.68 (1.08, 2.59)		1.45 (0.90, 2.33)	
Maintenance regimen		<0.001		<0.001
°°°Tacrolimus-containing	Reference		Reference	
°°°Cyclosporin A-containing or other	2.62 (1.63, 4.19)		2.83 (1.73, 4.65)	
Type of donation (living)	2.68 (1.46, 4.90)	0.001	4.77 (1.92, 11.85)	0.002
Donor age (per ten year increase)	1.08 (0.96, 1.21)	0.20	1.19 (1.05, 1.36)	0.007
Cold ischemia time (per ten minutes)	1.00 (0.99, 1.02)	0.85	1.02 (1.00, 1.03)	0.079
Duration of transplant surgery (per hour)	1.00 (0.93, 1.08)	0.95	0.99 (0.91, 1.07)	0.78
SSI	3.19 (1.44, 7.06)	0.004	2.97 (1.32, 6.68)	0.02

Abbreviations: HR hazard ratio, 95% CI 95% confidence interval, MELD model for end-stage liver disease

© 2025 Schreiber PW et al.

eTable 6: Sensitivity analysis for cause-specific Cox proportional hazard models for risk of death and/or graft loss in the first year after liver transplantation with nonlinear effects for numerical variables via restricted cubic splines treating surgical site infection as time-dependent

	Death/graft loss			
	univariable		multivariable	
**Characteristic**	**HR (95%CI)**	**p**	**HR (95%CI)**	**p**
Recipient sex (male)	0.89 (0.64, 1.24)	0.48	1.06 (0.72, 1.55)	0.78
Recipient age*		0.80		0.31
°°°20	1.01 (0.42, 2.45)		0.92 (0.34, 2.48)	
°°°30	0.98 (0.59, 1.64)		0.98 (0.55, 1.73)	
°°°40	0.97 (0.80, 1.17)		1.02 (0.83, 1.27)	
°°°50	Reference		Reference	
°°°60	1.09 (0.75, 1.58)		0.84 (0.55, 1.29)	
°°°70	0.82 (0.50, 1.35)		0.59 (0.34, 1.03)	
MELD score*		0.02		0.003
°°°10	0.97 (0.81, 1.17)		0.92 (0.75,1.12)	
°°°15	Reference		Reference	
°°°20	0.75 (0.56, 1.00)		0.78 (0.57,1.07)	
°°°25	0.60 (0.39, 0.92)		0.62 (0.39, 0.98)	
Induction immunosuppression		0.01		0.09
°°°Basiliximab	Reference		Reference	
°°°Antithymocyte globulin (ATG) or other induction therapy	1.54 (1.09, 2.18)		1.43 (0.95, 2.13)	
Maintenance regimen		<0.001		<0.001
°°°Tacrolimus-containing	Reference		Reference	
°°°Cyclosporin A-containing or other	2.95 (2.05, 4.23)		3.63 (2.46, 5.35)	
Type of donation (living)	2.43 (1.49, 3.97)	<0.001	5.74 (2.66, 12.40)	<0.001
Donor age*		0.48		0.09
°°°20	1.63 (0.86, 3.11)		1.86 (0.87, 4.00)	
°°°30	1.26 (0.90, 1.15)		1.45 (0.98, 2.14)	
°°°40	1.04 (0.88, 1.22)		1.17 (0.98, 1.40)	
°°°50	Reference		Reference	
°°°60	1.09 (0.83, 1.44)		0.91 (0.67, 1.24)	
°°°70	1.10 (0.78, 1.57)		0.85 (0.57, 1.26)	
Cold ischemia time (per ten minutes)	1.00 (0.99, 1.01)	0.75	1.30 (1.06, 1.60)	0.02

© 2025 Schreiber PW et al.

Duration of transplant surgery (per hour)	0.99 (0.92, 1.05)	0.61	0.88 (0.67, 1.13)	0.31
SSI	3.20 (1.83, 5.62)	<0.001	3.30 (1.85, 5.89)	<0.001

Abbreviations: HR hazard ratio, 95% CI 95% confidence interval, MELD model for end-stage liver disease

* Recipient age, MELD score and donor age were coded as restricted cubic splines.

© 2025 Schreiber PW et al.

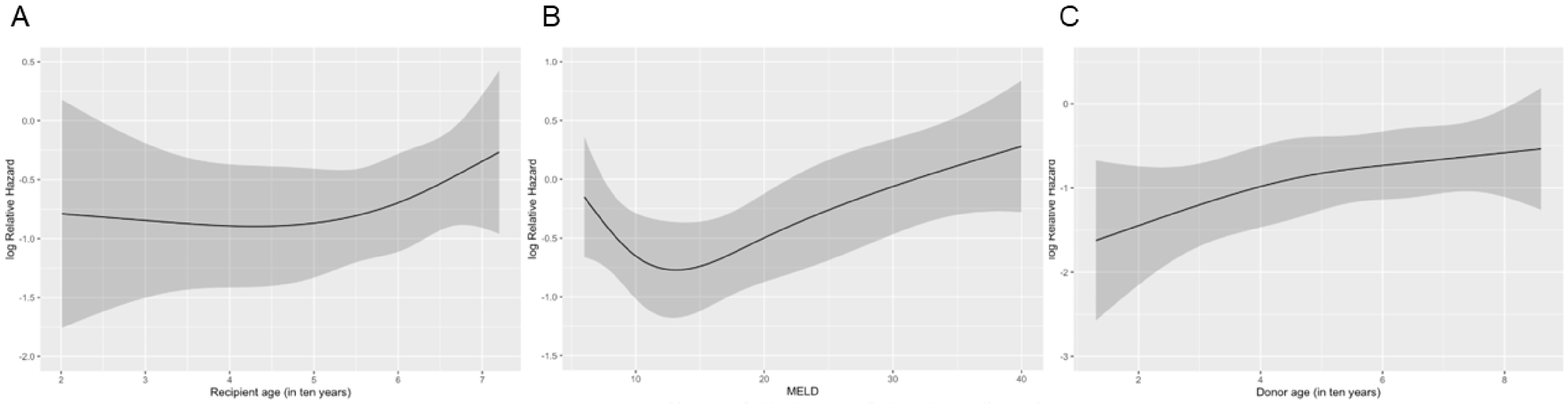

A, Association between recipient age and estimated hazard for the combined endpoint death/graft loss. The model is adjusted for the following variables (set at representative levels): sex, MELD score, induction immunosuppression, maintenance immunosuppression, type of donation, donor age, cold ischemia time, duration of transplant surgery, surgical site infection.
B, Association between MELD and estimated hazard for the combined endpoint death/graft loss. The model is adjusted for the following variables (set at representative levels): sex, recipient age, induction immunosuppression, maintenance immunosuppression, type of donation, donor age, cold ischemia time, duration of transplant surgery, surgical site infection.
C, Association between donor age and estimated hazard for the combined endpoint death/graft loss. The model is adjusted for the following variables (set at representative levels): sex, recipient age, MELD score, induction immunosuppression, maintenance immunosuppression, type of donation, cold ischemia time, duration of transplant surgery, surgical site infection.

The shaded area corresponds to the 95% confidence interval from the restricted-cubic-spline model.

eFigure: Visualization of recipient age/MELD/donor age-hazard ratio relationships from Cause-specific Cox proportional hazards model for risk of death and/or graft loss in the first year after liver transplantation corresponding to **eTable 6**

© 2025 Schreiber PW et al.
