# Supplementary material for: Surgical Site Infections, Risk Factors, and Outcomes After Liver Transplant
Source: JAMA Netw Open. 2025 Mar 21;8(3):e251333. doi: 10.1001/jamanetworkopen.2025.1333 (PMC11929024; doi:10.1001/jamanetworkopen.2025.1333)
Supplement: Supplement 2. — Nonauthor Collaborators. The Swiss Transplant Cohort Study [file jamanetwopen-e251333-s002.pdf]

Supplemental Online Content: Nonauthor Collaborators
*First name, last name, and suffix (if applicable) are required and will appear in PubMed.

*Group Name(s): Swiss Transplant Cohort Study							
***First Name and Middle Initial(s)**	***Last Name**	***Suffix (eg, Jr, III)**	Academic Degrees	Institution	Location (city, state/province, country)	Role or Contribution, eg, chair, principal investigator	Group (if more than 1 Group listed in the byline) and/or Subgroup (eg, Steering Committee)
Patrizia	Amico						
Adrian	Bachofner						
Vanessa	Banz						
Sonja	Beckmann						
Guido	Beldi						
Christoph	Berger						
Ekaterine	Berishvili						
Annalisa	Berzigotti						
Isabelle	Binet						
Pierre-Yves	Bochud						
Sanda	Branca						
Anne	Cairoli						
Emmanuelle	Catana						
Yves	Chalandon						
Sabina	De Geest						
Sophie	De Seigneux						
Joëlle L	Dreifuss						
Michel	Duchosal						
Thomas	Fehr						
Sylvie	Ferrari-Lacraz						
Jaromil	Frossard						
Déla	Golshayan						
Nicolas	Goossens						
Fadi	Haidar						
Dominik	Heim						
Christoph	Hess						
Sven	Hillinger						
Hans H	Hirsch						
Patricia	Hirt						

Supplemental Online Content: Nonauthor Collaborators
*First name, last name, and suffix (if applicable) are required and will appear in PubMed.

***First Name and Middle Initial(s)**	***Last Name**	***Suffix (eg, Jr, III)**	Academic Degrees	Institution	Location (city, state/province, country)	Role or Contribution, eg, chair, principal investigator	Group (if more than 1 Group listed in the byline) and/or Subgroup (eg, Steering Committee)
Linard	Hoessly						
Günther	Hofbauer						
Uyen	Huynh-Do						
Nina	Khanna						
Michael	Koller						
Andreas	Kremer						
Thorsten	Krueger						
Christian	Kuhn						
Bettina	Laesser						
Frédéric	Lamoth						
Roger	Lehmann						
Alexander	Leichtle						
Oriol	Manuel						
Hans-Peter	Marti						
Michele	Martinelli						
Valérie	McLin						
Katell	Mellac						
Aurélia	Mercay						
Karin	Mettler						
Nicolas	Müller						
Ulrike	Müller-Arndt						
Mirjam	Nägeli						
Graziano	Oldani						
Manuel	Pascual						
Rosemarie	Pazeller						
Klara	Posfay-Barbe						
David	Reineke						
Juliane	Rick						
Simona	Rossi						
Fabian	Rössler						
Silvia	Rothlin						

Supplemental Online Content: Nonauthor Collaborators
*First name, last name, and suffix (if applicable) are required and will appear in PubMed.

***First Name and Middle Initial(s)**	***Last Name**	***Suffix (eg, Jr, III)**	Academic Degrees	Institution	Location (city, state/province, country)	Role or Contribution, eg, chair, principal investigator	Group (if more than 1 Group listed in the byline) and/or Subgroup (eg, Steering Committee)
Frank	Ruschitzka						
Thomas	Schachtner						
Stefan	Schaub						
Dominik	Schneidawind						
Macé	Schuurmans						
Simon	Schwab						
Thierry	Sengstag						
Frederico	Simonetta						
Jürg	Steiger						
Guido	Stirniman						
Ueli	Stürzinger						
Christian	Van Delden						
Jean-Pierre	Venetz						
Jean	Villard						
Julien	Vionnet						
Caroline	Wehmeier						
Madeleine	Wick						
Markus	Wilhelm						
Patrick	Yerly						
