# Supplementary material for: Surgical Site Infections, Risk Factors, and Outcomes After Liver Transplant
Source: JAMA Netw Open. 2025 Mar 21;8(3):e251333. doi: 10.1001/jamanetworkopen.2025.1333 (PMC11929024; doi:10.1001/jamanetworkopen.2025.1333)
Supplement: Supplement 3. — Data Sharing Statement [file jamanetwopen-e251333-s003.pdf]

Data Sharing Statement

Schreiber. Surgical Site Infections, Risk Factors, and Outcomes After Liver Transplant. *JAMA Netw Open*. Published March 21, 2025. doi:10.1001/jamanetworkopen.2025.1333

Data
Data available: Yes
Data types: Deidentified participant data
How to access data: www.stcs.ch
When available: With publication

Supporting Documents
Document types: None

Additional Information
Who can access the data: Data can be requested for scientific purposes after approval by the STCS Scientific Committee and the corresponding Ethics Committees.
Types of analyses: For specified purposes
Mechanisms of data availability: After approval of a proposal by the STCS Scientific Committee.
Any additional restrictions: After approval by the corresponding Ethics Committees.
